# Moral Agency, Rules, and Temporality in People Who Are Diagnosed With Mild Forms of Autism: In Defense of a Sentimentalist View

**DOI:** 10.3389/fpsyg.2022.875680

**Published:** 2022-06-28

**Authors:** Sara Coelho, Sophia Marlene Bonatti, Elena Doering, Asena Paskaleva-Yankova, Achim Stephan

**Affiliations:** ^1^Faculty of Medicine, University of Lisbon, Lisbon, Portugal; ^2^Department of Psychology and Cognitive Science, University of Trento, Rovereto, Italy; ^3^German Center for Neurodegenerative Diseases (DZNE), Bonn, Germany; ^4^Department of Nuclear Medicine, University Hospital Cologne, Cologne, Germany; ^5^Institute III, Philosophy, Otto-von-Guericke-University Magdeburg, Magdeburg, Germany; ^6^Institute of Cognitive Science, Osnabrück University, Osnabrück, Germany

**Keywords:** autism spectrum disorder, empathy, moral agency, (moral) rationalism, (moral) sentimentalism, temporality

## Abstract

The origin of moral agency is a much-debated issue. While rationalists or Kantians have argued that moral agency is rooted in reason, sentimentalists or Humeans have ascribed its origin to empathic feelings. This debate between rationalists and sentimentalists still stands with respect to persons with mental disorders, such as individuals diagnosed with mild forms of Autism Spectrum Disorder (ASD), without intellectual impairment. Individuals with ASD are typically regarded as moral agents, however their ability for empathy remains debated. The goal of this paper is to investigate the mechanisms of moral actions in people with ASD, by finding arguments for the origin of their moral actions, supporting either the sentimentalist or the rationalist view of the dispute. We propose to revisit the debate using Interpretative Phenomenological Analysis to study the autobiographies of individuals with High-Functioning Autism (HFA) and Asperger Syndrome (AS). While conducting the systematic analysis of 10 autobiographies, we re-examined both the rationalist and the sentimentalist positions, considering the links between empathic feelings and moral agency. The investigation of the temporal dimensions of emotional experiences, an aspect overlooked by previous research, indicated that individuals with ASD empathize with others, but in different ways as compared to neurotypicals. A relationship between emotional experience and the type of moral agency exhibited by individuals with forms of ASD was established. As a consequence, our analyses support the sentimentalist stance on moral action.

## Introduction

Studying the origins of moral agency^1^ is important in many practical respects. For instance, developmental psychologists are interested in information useful in the education of moral agency, and medical experts and therapists deploy knowledge about moral agency to help people with moral deficits (Muhaimin et al., 2020). The quest for the roots of moral agency has always occupied philosophers from different traditions and leanings. Rationalists or Kantians have defended the view that moral actions are attributable to reason while sentimentalists or Humeans have argued that moral actions stem from empathic feelings (McGeer, 2007). Nowadays, many philosophers (e.g., Saunders, 2016) and neuroscientists (e.g., Greene, 2008) support the view that both rational and emotional processes are interwoven in moral agency. However, the debate between rationalists and sentimentalists has not been resolved with respect to populations with mental disorders. Studying the moral actions of a group of individuals who exhibit differences from the neurotypical population in affective and reasoning capacities can potentially help elucidating whether each of these capacities alone suffices for moral agency. At the same time, an answer to this question, based on the investigation of the experiences and actions of people with mental disorders may shed new light upon this old philosophical controversy, favoring a rationalist or a sentimentalist position in view of neuroatypical individuals (Kennett, 2002; McGeer, 2007; Bollard, 2013).

A recent focus of the debate between rationalists and sentimentalists has concentrated on questions about the mechanisms of moral agency, informed by empirical evidence from case-studies of individuals with High-Functioning Autism (HFA) and Asperger Syndrome (AS) (Kennett, 2002; McGeer, 2007; Bollard, 2013). The fact that the empathic experience of these individuals differs from that of neurotypicals raised questions about the impact of emotional experience upon moral actions, putting into question the sentimentalist position. While the studies of Bollard, Kennett, and McGeer offer invaluable insights, it is important to note that they do not engage in a systematic examination of autobiographical accounts. Individual passages from first-person accounts are taken to illustrate important aspects of the origins of moral agency that either sustain or contradict the sentimentalist view [see, respectively, Kennett (2002) and Bollard (2013)].

Motivated by these considerations, we engaged in the systematic examination of several first-person accounts of individuals with HFA and AS specifically in view of their reports and descriptions of moral agency, rational reasoning, and experiences of empathy [similar to the studies conducted by Williams (2009) and Elwin et al. (2012)]. In doing this, we discovered what we consider to be a neglected, yet important relationship between the temporal dimensions of emotional experience and empathy that may further inform mechanisms of moral agency. We show that the examination of the relationship between temporality and empathy in persons with HFA and AS helps us understand the specificities of their moral actions and, thus, provides further support for the sentimentalist position. Moreover, we suggest that the examination of first-person accounts and the insights into the mechanisms of moral agency can be used as the basis for designing specific empirical measures for assessing moral actions.

Another point worth mentioning is that the majority of the authors considered in our analysis were diagnosed in the nineties of the 20th century or in beginning of the 21st century, having spent their childhood and teenage years undiagnosed or misdiagnosed. These circumstances may have impacted the expression of the respective disorders by leading people with ASD to reacting in peculiar ways, designing, and applying particular strategies to fit into society. Two consequences of this stand out. On the one hand, the knowledge of the disorder at the time of the diagnosis is not the same as nowadays, thus the best treatment may not have been provided. On the other hand, not having received a correct diagnosis has led people with ASD to be disregarded in their needs and not be understood by the world surrounding them.

## High Functioning Autism, Asperger Syndrome, and Empathy

Autism spectrum disorder is a complex neurodevelopmental disorder characterized by persistent impairments in social communication and social interaction across multiple contexts, as well as by the presence of restricted, repetitive patterns of behavior, interests, or activities (American Psychiatric Association, 2013). HFA and AS are presently considered as mild forms of autism in the Autism Spectrum Disorder^2^ continuum (DSM-5, 2013, p. xlii). Persons diagnosed with mild forms of the spectrum possess a normal or high IQ (they are not cognitively impaired) but experience social and communicational problems (Gillet, 2014).

Although persons suffering from ASD perform moral actions (Smith, 2009), there has been no consensus yet on whether they lack empathy (Smith, 2009; Gillet, 2014). Some empirical studies relate facial emotional recognition with empathy, postulating that the difficulty to identify emotions in others’ facial expression may be an indicator of difficulties in emotional sharing (Gillet, 2014; Loth et al., 2018). Other researchers have argued for a more differential view, claiming that individuals with ASD may present a deficit in *cognitive empathy* while their *affective empathy* is preserved (Smith, 2009). In fact, another study points to a correlation between facial emotional recognition and cognitive empathy, but not affective empathy (Liu et al., 2016). It is important to note that Liu et al. (2016) showed that higher levels of callous-unemotional traits, such as lack of guilt, were associated with a relative deficit in affective empathy (but not with cognitive empathy), when partially mediated by affective perspective taking (“the ability to infer emotional states of other”).

Cognitive empathy refers to the ability to understand others’ emotions, beliefs or motives such as when e.g., Mary *knows* that Peter is in despair because he went bankrupt. On the other hand, affective empathy refers to the ability to share another person’s affective state by observing or imagining it (De Vignemont and Singer, 2006; Paulus et al., 2013): e.g., when Mary looks at Peter’s face, covered in tears, and *feels* sad. Affective empathy can lead empathizers to exhibit emotions of distress and compassion toward another person’s affective state (Rogers et al., 2007), which indicates experiencing a concern for the other^3^ : E.g., when Mary is feeling sad at Peter’s despair and is, thus, concerned about him.

Previous studies have shown that people with ASD demonstrate moral agency, as they exhibit signs of distress and compassion toward other people’s suffering (Jones et al., 2010; Hadjikhani et al., 2014). Feeling others’ emotions seems to prevent them from harmful behavior (Jones et al., 2010). However, people with ASD are assumed to be incapable of understanding the motives, thoughts, and intentions behind others’ suffering (Smith, 2009). Some researchers sustain the view that people with ASD produce more outcome-based decisions rather than intent-based decisions, due to difficulties attributing intentions to others (see Dempsey et al., 2020). Interestingly, in other pathologies such as psychopathy,^4^ individuals have only cognitive empathy, that is, they understand others’ motives and beliefs, but lack moral agency. According to some studies, people with psychopathy are capable of representing their own emotions when imagining themselves in another’s place (that’s how they come to *understand* emotions), although they remain impaired at simulating others’ emotions in themselves (Decety et al., 2013; Bird and Viding, 2014). Thus, they lack compassion or concern and sometimes may even harm other people (Blair, 2007).

## The Rationalism-Sentimentalism Debate Using Case-Studies of People With Autism Spectrum Disorder

Using case-studies that present how individuals with ASD perform moral reasoning, Kennett (2002) fostered the rationalist position with respect to the origins of moral agency in people with ASD. She argued that empathy can neither be a driving moral force in people with ASD nor in persons with psychopathy because both lack empathy in some ways. As previously stated, while persons with psychopathy are amoral, individuals with ASD are moral. Contrary to psychopaths, who reportedly are indifferent to others’ rights or needs, individuals with ASD show concern and caring behavior (Blair, 2007; Jones et al., 2010; Hadjikhani et al., 2014). Thus, according to Kennett, the lack of full-fledged empathy, at least by itself, cannot explain the lack of moral agency. Persons with ASD are committed to doing the right thing, acting by duty to that imperative and, in the absence of both empathy and negative feelings toward others, create rules for acting, which are based on reason (Kennett, 2002). To illustrate her view, Kennett gave the example of Jim Sinclair, a person suffering from autism. After seeing a person sobbing, Sinclair started to reason what he should do and decided to touch her. Thereby, he formulated a rule to himself that when he sees a person crying, he/she must be touched for comfort.^5^

In the years to follow, Kennett’s position was subjected to criticism. Bollard (2013) used Sinclair’s example to sustain a sentimentalist position referring to the source of moral agency in people with ASD. Drawn on Sinclair’s example, her view is that people with autism, unlike psychopaths, have affective empathy, which provides them with tools for moral understanding. That moral understanding, in turn, leads to the creation of rules, which invoke moral actions. For instance, when Sinclair observed someone sobbing, he would affectively empathize with her despair and attempt to act appropriately to the situation. It was his affective empathy, though, that guided him in creating the rule to determine his action. Although Bollard argued for a sentimentalist position, she nonetheless preserved some of Kennett’s insights, namely that obedience to rules guides the moral actions of individuals with ASD. In current perspectives, neurotypical individuals require both reasoning and emotional processing to produce moral actions (Greene, 2008). Findings from neuroscience indicate that moral decision making involves the prefrontal cortices, which are dedicated to reasoning, as well as the insula and the posterior cingulate, which are involved in emotional processing (Pascual et al., 2013). Thus, both rational and emotional processing contribute to the development and exercise of moral agency. Individuals with ASD exhibit difficulties in both domains but nevertheless produce moral actions. It is also claimed that they process moral information differently when making moral decisions (Pascual et al., 2013). In the upshot, the sentimentalist position argues that emotions suffice to produce a moral response, while the rationalist position sustains that reason alone is sufficient.

Whether it is rules that govern the moral actions of individuals with ASD, as suggested by Kennett (2002) and Bollard (2013), is also subject to debate. Some authors sustain that our moral considerations come from intuition, prior to any reasoning (Haidt, 2001). Others suggest that individuals with ASD can prioritize specific moral foundations (Dempsey et al., 2020), such as authority and rules (Fadda et al., 2016; Margoni and Surian, 2016; Bellesi et al., 2018). However, De Vignemont (2007) has argued that it is difficult to understand if people with autism are following rules because they genuinely think that these norms should apply to everybody (universal rules) or because they are merely adapting to the conventions of the external world. At the same time, the researcher added that affective empathy does fully account for the creation of such norms, an opinion that is shared by McGeer (2007). While McGeer (2007) agrees with Kennett (2002) in affirming the fact that people suffering from ASD privilege a life guided by rules and empathy does not explain moral actions, she refuses to share Kennett’s conclusion that reverence to reason is the core moral motive. By being educated within certain rules, people with ASD develop moral sensibility that leads them to favor certain rules and not others. Due to affect, some rules appear to be more important than others. Understandably, difficulties in emotional identification (namely alexithymia, which in many cases co-occurs with autism; Bird and Cook, 2013) make the range of emotions, which support their choices, differ from that of neurotypicals. In the case of alexithymia, for instance, one might not be able to cognitively empathize with all emotions others manifest, as there might be difficulties identifying at least some of them (Goerlich, 2018).

In our opinion, neither Kennett (2002) nor her critics (McGeer, 2007; Bollard, 2013) gave enough evidence for capturing and explaining the degree of complexity that the moral actions of people with ASD show. It is important to understand the ways in which individuals with ASD deal with rules and if their moral actions reflect solely obedience to rules or if they are the product of peculiar emotional experiences in order to be able to draw any conclusions about the origins of their moral actions. In order to do this, we engaged in a detailed and systematic analysis of first-person accounts by individuals with ASD, including also an examination of a so-far overlooked relationship between emotions, temporal experience, and moral reasoning/behavior that can shed light on the complexity of the moral agency of individuals with ASD.^6^

## The Role of Temporal Experience in Emotion

We consider temporality as the experience of temporal flow that is implicit in all thinking, feeling, and doing, i.e., as temporal consciousness that is constantly pre-reflectively present. In the phenomenological literature, this kind of temporal experience has also been labeled “implicit temporality” (Fuchs, 2005, 2013; Fuchs and Pallagrosi, 2018) and is commonly altered in various psychopathological conditions [see Fuchs (2013) for a discussion of disturbances of temporality in psychopathology]. In contrast to it, explicit time is the reflective experience of time as, for instance, in cases of autobiographical recollection (Fuchs and Pallagrosi, 2018). This latter form of temporality is not in the focus of our work, as it more closely relates to, for instance, a narrative conception of the self, deliberate (autobiographical) recollection, etc. rather than lived time. The experience of lived time or implicit temporality is an essential feature of subjectivity and a prominent theme in the works of Husserl, Binswanger, and Minkowski just to name a few of the most comprehensive and influential classical accounts. Recently, the study of temporality from a phenomenological perspective, especially in terms of its disturbances in cases of psychopathology, has also received substantial attention [e.g., in the works of Ratcliffe (2012); Fuchs (2013), and Fuchs and Pallagrosi (2018)] and in cognitive science (a discussion of different accounts can be found in Dainton, 2018). Building on Husserl’s work, Fuchs (2005, 2013) and Fuchs and Pallagrosi (2018) view implicit time as the synthesis of the just past moments and the present that extends into the future. Thereby, when we have a particular experience, it contains not only an impression of the present moment (Husserl’s presentation) but also of the recently past moments (Husserl’s retention) and an anticipation of what is to come (Husserl’s protention) and provides us with a sense of continuity and coherence of subjectivity over time. For instance, witnessing a vase falling from a shelf, my awareness of this present moment contains also the impression of the vase being pushed by my arm and a general anticipation of it continuing to fall, hitting the hard floor, and possibly shattering into pieces. The experience of lived time, it has been suggested (Fuchs and Pallagrosi, 2018), has a conative dimension as well, which has energizing potential that moves one to act. In the aforementioned example, in anticipating the fall and shattering of the vase, I might be affected and moved to reach out for it and grab it, to perform a particular action that is in line with the anticipated consequences of the witnessed event.

As a fundamental aspect of subjectivity, implicit temporality figures in both the experience of one’s own emotions and in empathizing with the emotions of others. When we experience affective empathy, we come to share other persons’ emotions in several ways. The empathic arousal modes register the experience of time passing (Hoy, 2009) and involve different cognitive processes (Davis, 2018). We may directly perceive other persons’ emotions by having a present and immediate experience of them. This stage corresponds to what Hoffman called *conditioning and motor mimicry* and happens when someone feels distress at others’ suffering (Hoffman, 1978): e.g., Mary sees Peter sobbing and feels sad. In other circumstances, the empathizer may experience the other persons’ affective state by a simple cognitive process of associating the emotions presently felt with his own past emotional experience (Wondra and Ellsworth, 2015), and thus establishing a connection between the past and the present. Hoffman called this phase *reflexive cry* (Hoffman, 1978): E.g., Mary sees Peter sobbing and feels sad, while remembering the last time she felt sad. In certain contexts and relationships, the empathizer may, in a higher cognitive process, even link the emotions felt at the present moment with his past experience and anticipate people’s future reactions and predict future emotions (Izard, 2009), linking the three temporal dimensions. Hoffman named this stage *imagining oneself in the other’s place* (Hoffman, 1978). E.g., Mary sees Peter sobbing, feels sad, while recalling the last time she felt this way and imagines that Peter will despair, and possibly commit suicide.

It has been suggested that these empathic arousal modes foster compassionate behavior (Hoffman, 1984). However, depending on the elicited empathic arousal mode, a subject may engage in more or less complex behavior, from simple empathic distress to advanced moral judgments (Hoffman, 1977). For instance, at the *conditioning and motor mimicry* stages, the subject may experience empathic distress and directly feel guilty for others’ suffering. In addition, at the *reflexive cry* phase, the observer may feel guilt over inaction and arrive at the moral judgment and blame herself for not having done anything to alleviate the other’s misery. Lastly, at the stage of i*magining oneself in the other’s place*, the subject may go through feelings of anticipatory guilt, where she may foresee probable distress responses to the other’s imagined acts (Hoffman, 1978). An example of a more advanced moral judgment would be to relate Peter’s love and jealousy toward Mary having a boyfriend with his subsequent behavior, which is aimed at separating Mary from her boyfriend.

## Hypotheses

As already mentioned, recent philosophical works have studied the moral actions of populations with mental disorders by recurring to the rationalism-sentimentalism debate. By doing so, they have utilized the analysis of autobiographical reports describing the affective experience of people with HFA and AS, as supporting both theoretical sides. Nevertheless, to the best of our knowledge, this discussion has not yet been informed by a systematic analysis of such reports that also addresses the complexity of affective experience. The analysis of first-person reports of affective experience may indeed provide invaluable insight into the subjective experience of people with AS and HFA, reappraising the controversy. To do this, we engaged in a systematic study of first-person reports by first examining how persons with AS and HFA behave toward rules. Secondly, we proceeded to a study of their emotional lives, and ultimately, we assessed the influence temporal dimensions of emotional experiences have on (moral) behavior.

Previous clinical and empirical studies pointed out that people with ASD have a different intuitive sense of time, exhibit difficulties experiencing the connections between temporal frames (Boucher, 2001) and focus on factual aspects of time rather than lived ones (Zukauskas et al., 2009). Interestingly, these lived aspects of time and its relationship with emotional experience in subjects with HFA and AS have not been studied sufficiently (Falter and Noreika, 2011; Vogel et al., 2018). Individuals suffering from HFA and AS exhibit signs of moral sensibility by showing concern, such as seeing a person crying and wishing to mitigate that pain. However, they encounter difficulties with respect to morally complex behaviors, such as making appropriate moral judgments, which involve following a temporal sequence from the agent’s intentions to the outcomes of the action (Senland and Higgins-D’Alessandro, 2013; Margoni and Surian, 2016). For instance, it would be difficult for them to relate, for example, Peter’s experience of sadness when he sees an empty spot previously occupied by a vase with the fact that he accidently broke it a week ago. Here the difficulty stands in relating Peter’s intentions (to not break the vase) with the outcomes of the actions, namely the broken vase and his sadness that he experiences again after the event. A sense of the extension of temporal flow into a future moment seems to be altered that may be associated with problems relating past and present moments with future ones, as in this example. The pre-reflective integration of past, present, and future captured by the notion of implicit time underlies also complex anticipations of the consequences of one’s intentions and actions that we constantly perform in everyday life.

Based on the difficulties of persons with autism to follow a temporal progression, presented by previous empirical studies, we expect to find, in this study, evidence that it is the experience of *immediate* empathic feelings that leads persons with HFA and AS to performing moral actions. Such empathy-guided moral agency would strengthen the sentimentalist stance on moral agency.

## Methodology

### Autobiographical Material Studied and Its Selection Criteria

Two groups of criteria were used to select the autobiographical material. The first centered on criteria pertaining to the quality of the sources used. As suggested by Scott (1990), we focused on authenticity, credibility, representativeness, and meaning in choosing the autobiographical works we assessed. This resulted in choosing a total of 23 autobiographies from those listed on the websites Neurodiversity.com and autism.org.uk. Consequently, the origin of the sources should be unquestionable, the sources should be free from errors or distortions, the texts selected should represent typically the lives of people with HFA and AS and the texts should be clear and comprehensible (Ahmed, 2010). We addressed the first two issues by choosing books written by a single author each and not biased by comments of a third person. We chose authors who are well-known persons with AS and HFA to ensure representativeness. The meaning of the texts emerged as clear and straightforward.

The second group of criteria was whether the autobiographies mentioned aspects such as temporality, empathy, moral agency, emotions, and rules. By using this latter guideline, nine autobiographies were selected from the initial sample. After exhausting the search in the two databases mentioned above, a tenth autobiography was chosen by setting the words autobiography and autism in google.com and following the two groups of criteria.

The resulting sample presents authors from different nationalities (the United States of America, the United Kingdom, Australia, Germany, and Sweden) who have either the diagnosis of High Functioning Autism (four) or of Asperger Syndrome (six). All authors show a high level of education and are adults. A limitation stands out as the sample has four times more women than men (please see Table 1 for an overview of the information about the sources and their authors).

**TABLE 1:** Diagnosis, time of the diagnosis, gender, nationality, and education of the authors at the time they first published their autobiographies.

Author	Title	Gender	Diagnosis	Date of publication (1st edition)	Time of the diagnosis	Nationality	Education
Edgar Schneider	Discovering my Autism	M	HFA	1999	1995	German	BSC
Laura James	Odd Girl Out	F	AS	2017	2015	British	BSC
Elkie Kammer	Discover who I am	F	AS	2007	2000	German	BSC
Wendy Lawson	Life behind a Glass	F	AS	1998	1994	Australian	PhD
Liane Holliday Willey	Pretending to be Normal	F	AS	1999	1999	British	PhD
Gunilla Gerland	A Real Person	F	HFA	1996	1992	Swedish	BSC
Dawn Prince-Hughes	Songs of the Gorilla Nation	F	AS	2004	2000	American	PhD
John Elder Robinson	Look me in the Eye	M	AS	2007	1997	American	High School
Donna Williams	Nobody Nowhere	F	HFA	1992	1991	Australian	BSC
Temple Grandin	Thinking in Pictures	F	HFA	1995	1986	American	PhD

### Interpretative Phenomenological Analysis

The particular methodology chosen for studying the autobiographic accounts was the Interpretative Phenomenology Analysis (IPA), as described by Smith et al. (1999). While reading the autobiographies of individuals with ASD, the researcher records descriptions of these self-reports and later organizes them in themes and sub-themes according to the purposes of the investigation. The goal is to discover common features between different reports (Smith et al., 1999). The advantage of this methodology over a more strongly structured analysis that is guided by the search for pre-defined specific categories and sub-categories, is that the former is better suited for understanding the lived experience of individuals with ASD (Smith et al., 1999). Delving into the subjective experience of individuals with HFA and AS is relevant to assessing how they experience emotions and relate to time and rules.

The ten autobiographical accounts of individuals diagnosed with HFA and AS were, in line with the research aims of this study, analyzed for recurrent descriptions of five predetermined major topics, essential to the aims of the research: rules, emotions, empathy, time, and moral. Themes emerged within single narratives and upon comparison among these. The method for identifying those themes followed several steps. At first, the autobiographies of two well-known writers with ASD were assigned to a group of researchers. Two researchers worked on the book *Real Person* by Gunilla Gerland (1997) and one of them studied with a further researcher *Life behind the Glass* by Wendy Lawson (2000). Each researcher first selected the passages related to the predetermined major topics. Anything significant described in the selected passages was annotated in the left margin. Then, the chapters were re-read and the researchers independently of each other looked for commonalities between the annotations, so that any emergent theme could be identified and annotated in the right margin. Later, each investigator compared their annotations with those of the other colleague. Agreement between researchers was high, meaning most of the themes recorded in the individual analysis matched each other. A final agreement on the list of themes and subthemes was reached between all researchers concerning the above-mentioned books and two chapters of four other books, *Pretending to be Normal* by Willey (1999), *Thinking in Pictures* by Grandin (2006), *Discovering who I am* by Kammer (2007), and *Look me in the eye* by Robinson (2008).

One researcher (Sara Coelho), then, completed the IPA of the four aforementioned books and analyzed four other publications*: Odd girl* by James (2017), *Nobody Nowhere* by Williams (1999), *Songs of a Gorilla Nation* by Prince-Hughes (2004), and *Discovering my Autism* by Schneider (1999). She used the themes and subthemes agreed with the group as guidance to perform the remaining analysis. Rigor in the qualitative analysis was established by the agreement of all three researchers on the themes that were constantly discussed in the process of analyzing the autobiographies. The final analysis was approved by all group members. The list of themes and sub-themes chosen for this article were selected according to their frequency within and between narratives.

Utilizing idiographic approaches such as IPA, it can be argued, goes along with certain limitations pertaining to generalizing to diverse populations on the basis of a small number of testimonies that reflect the experience of their authors only. This is commonly acknowledged in the research of autobiographical accounts of individuals with autism. Davidson and Smith (2009), for instance, argue that while the study of only a limited number of autobiographical accounts can, for various reasons, present us with serious limitations, we can nonetheless generalize our findings. According to them, “autobiographical writings constitute uniquely suitable “data” for qualitative research on autism” (Davidson and Smith, 2009, 902). It should be noted that the majority of the authors of first-person accounts are women who, moreover, are at the higher end of the spectrum. While this is a limitation that we acknowledge, it does not entirely preclude the generalization of our findings to the diverse populations of individuals with Autism spectrum disorder because as a spectrum disorder autism comes in different forms that do share phenomenological similarities (Davidson and Smith, 2009). Thus, while our findings might not be representative of each and every individual on the autistic spectrum, they provide insights into the first-personal experience of autism.

One of the advantages of IPA, as pointed out above, is that it provides access to the experiential dimension of one’s existence. Through the narratives analyzed we can observe how external events impact the inner lives of individuals with ASD, how those are experienced and how the environment influences the development of their condition.

## Rules, Emotions, and Temporality

Three main themes emerged from our interpretative phenomenological analysis, revolving around how individuals with HFA and AS deal with rules, what their emotional life is, and how temporality relates to their emotional life. These themes present us with descriptions of (1) difficulties, or even a refusal to following universal rules, of (2) a peculiar emotional life characterized by a specific range of affective experience, and of (3) difficulties in connecting time frames or temporal dimensions. Each theme comprises several subthemes (see Table 2 for an overview of the themes and sub-themes).

**TABLE 2:** Themes and *sub-themes* (plus the number of authors that identified the sub-themes).

Themes and *sub-themes*
**Not following universal rules**
*Difficulty to generalize* (7/10)
*Using norms as techniques to thrive* (9/10)
*Norms only apply to themselves* (10/10)

**Feeling a specific set of emotions**
*Feeling basic but not complex emotions* (8/10)
*Compassion* (10/10)
*Affective empathy leads to moral action* (10/10)

**Difficulty to connect time frames**
*Predicting consequences/anticipate others’ behavior* (8/10)
*Imagining the future* (7/10)
*Feeling emotions at a particular temporal frame* (6/10)

### Following Rules

According to Kantian ethics or the rationalist position, subjects should pursue rules that their reason creates. Then, they should act as if they want these rules to apply to everyone, so-called universal rules. These might be taken to apply to everyone, as in basic moral principles or laws, for instance that one should not steal, or to all members of specific groups or involved in specific situations, such as the rule that everyone in the audience at the opera should not disrupt the performance. All authors examined reported that they design specific norms to act in the world or decide to adopt certain guidelines created by other people. However, the norms created by the authors with ASD neither apply to everybody nor are they valid in every situation. They tend to postulate norms specific to particular situations and particular persons. Therefore, individuals with HFA and AS do not obey by duty to a universal rule, which runs against Kennett’s (2002) explanation.

The lack of generalization of rules and their universal application was present in several different, yet closely related subthemes. For instance, Lawson explicitly emphasized the difficulty to draw generalizations over multiple situations or to apply rules in situations that differ from the one at hand as reported in the following passage and captured by the subtheme *difficulty to generalize*^7^ :

Autistic people find it very hard to generalize. They may learn the rules for a particular situation but will not know how to translate that into other situations (Lawson, 2000, 100).^8^

Individuals with HFA and AS experience some troubles in recognizing commonalities between past experiences and new ones. This seems to be linked to their difficulty in relating temporal dimensions or frames in a coherent sequence (reflected by the theme *difficulty to connect time frames*), which we will discuss later. Williams, for example, illustrates the difficulty to generalize rules or guidelines across various situations and the subsequent failure to adhere to these in different situational context as follows:

The significance of what people said to me, when it sank in as more than just words, was always taken to apply only to that particular moment or situation. Thus, when I once received a serious lecture about writing graffiti on Parliament House during an excursion, I agreed that I’d never do this again and then ten minutes later, was caught outside writing different graffiti on the school wall. To me, I was not ignoring what they said, nor was I trying to be funny: I had not done *exactly* the same thing as I have done before (Williams, 1999, 64).

What these passages poignantly describe runs counter to the universalization of rules proposed by Kennett. People with HFA and AS have difficulties creating a general rule that applies to even slightly different situations. Williams understood the interdiction of not writing graffiti. However, she internalized that prohibition only for the walls of the Parliament. She did not extend this rule to other situations or contexts such as those involving other buildings. She did not recognize the second situation as similar to the first situation.

The failure to universalize rules that guide one’s behavior across different situations is manifested in the reported motivation for creating these rules. Most of the authors mentioned that the norms they adopted are strategies to fit in society, designed to deal with the situations they face, so that they can thrive (subtheme *using norms as a technique to thrive*). This strategy of creating rules and following them would be adopted even in cases, in which they might conflict with values such as honesty, as one of the authors pointed out:^9^

As I got older, I found myself in trouble more and more for saying things that were true, but that people didn’t want to hear. I did not understand tact. I developed some ability to avoid saying what I was thinking (Robinson, 2008, 33).

The obedience to guidelines created by other people is rather a strategy to avoid problems. The authors do not take these criteria as internal commandments that should dictate everybody’s actions but as principles that help them navigate the world better as pointed out by Gerland:

In that teacher’s world, you simply couldn’t be as good at spelling as I was. The way she saw it, I was afraid of making mistakes, so I looked things up in the book when we had spelling tests. But the fact was that I would never even have dreamt of cheating. I always obeyed the rules made for me as long as they didn’t conflict with my most important needs […] I made an effort to make plausible mistakes, and I varied the number of mistakes between one and three each time. I thought that was just about right. The rules you lived in this world were very strange (Gerland, 1997, 93–94).

Committing some spelling mistakes, thus, is led by the wish to stay away from trouble and not because the rule itself is considered meaningful. This might result in a refusal to follow at least some specific universal rules (that is, rules that should be applied to everyone in particular situations) just because they conflict with one’s personal needs. For instance, not adhering to requirements as one might find these challenging, physically demanding, etc. can be interpreted as a reluctance or refusal to follow rules and guidelines that should guide the behavior or performance of everyone as explicitly elaborated in this passage:

At a certain age we were supposed to be able to turn somersaults, and I was the only one who couldn’t. When I got on all fours and put my head down, all sense of space, direction and body simply vanished. I tried my usual method of dealing with life’s difficulties – I refused. (Gerland, 1997, 113)

It might be argued that this tendency to create specific norms conflicts with the failure of predicting the consequences of one’s actions (which is captured by the subtheme *predicting the consequences*). If people with HFA and AS use norms as strategies to fare better in society or thrive, they may be able to predict the future. Nevertheless, if that statement was true, they would be at ease with generalizing, which, as already discussed, is not the case. It can be argued that the specific norms that people with ASD adopt in specific situations help them to solve their problems, in a word, to thrive. However, the solution is helpful in the short-term. The adoption of particular norms brings long-term negative consequences, for instance, it can make social integration more difficult.^10^

The norms individuals with HFA and AS use may also not be generalized across individuals but are taken to hold only in one’s own case. For instance, this failure might be reflected in the preference of one’s own rules that can be treated as exceptions as pointed out in the following passage and captured by the subtheme *norms only apply to themselves*^11^ :

Just later on at school, if I was ever granted special treatment and allowed to do something on my own, then I was sure to be made aware of the fact that it was a favor and that I should be grateful […] The few times I escaped to this kind of response, when someone just allowed me a favor without any special admonitions, I could almost feel the disapproval of other adults over how pampered I was (Gerland, 1997, 71–72).

Further instances of the failure to universalize rules across individuals can be seen in the reluctance or unwillingness to adopt the rules others are using and prefer one’s own, as in these instances:

For as far back as I can remember, I always had my own set of values […], and, as such, did not hesitate to question the values of others if they seemed to me to be illogical (One example is the questioning of my family’s values that eventually led to my adoption of Catholicism) (Schneider, 1999, 102).

However, there were many times when the company of the others became too much for me, especially, as I mentioned before, when the storyline of our adventures took on a path which I hadn’t expected and which didn’t seem logical to me. Then I preferred to withdraw, often to the annoyance of the others, to carry on my own version. I never understood why it made them angry, though. After all, I wasn’t interfering, just leaving them to their own devices (Kammer, 2007, 23).

Individuals with autism have values, as Schneider stated. Nevertheless, the fact that persons with HFA and AS embrace certain values does not mean that they believe that these values are universal. They feel entitled, like Kammer, to have their own particular codes of conduct and have trouble seeing those as shared by everybody. For instance, they adhere to the value “I should not steal” but have difficulty to cope with the principle “People should not steal.”

Individuals with HFA and AS have difficulties not only in generalizing but also in applying universal principles to concrete specific actions (Wass and Porayska-Pomsta, 2013). However, despite the aforementioned difficulties, it could be added that detailed attention to particular cases makes them better moral actors than neurotypicals when it comes to atypical cases. For instance, Gerland shined dealing with difficult children and the elderly because she notices things that nobody else notices. While she was working at an institution, she was the only one able to understand what an old lady with dementia wanted, by paying careful attention to her body language.

### Range of Affective Experience

A further aspect described in autobiographical accounts of individuals diagnosed with ASD that is of special relevance to our interests is their emotional life, which the theme *feeling a specific set of emotions* assesses. Within the framework of the rationalism-sentimentalism debate, Bollard (2013) has sustained, contrary to Kennett (2002) and De Vignemont (2007), the importance of affective empathy for performing moral actions. She argues that building rules is only possible thanks to moral understanding, provided by affective empathy. The previous theme has shown that although people with HFA and AS create norms to act, they do not follow universal rules. The descriptions of emotional experience and the own emotional repertoire, which are the subject of the current section, suggest that the norms they adhere to or create are not necessary for performing moral actions. The range of emotions they experience appears to differ from that of neurotypicals as the examined autobiographies include descriptions of experiencing only certain emotions (captured by the subtheme *feeling basic but not complex emotions*) and of experiencing compassion (reflected by the subtheme *compassion*). When it comes to actions properly labeled moral, people with HFA and AS rather than relying on norms that they create to strategically survive (and that in some cases conflict with inner values), seem directly compelled to behave morally. Most of the authors mentioned empathic feelings as motivating their moral actions, without the need to create norms (captured in the subtheme *affective empathy leads to moral actions*).

Some authors, particularly those with HFA, for example Grandin, reported being able to feel solely certain emotions, such as fear, anger, happiness and sadness^12^ :

My emotions are simpler than those of most people. I don’t know what complex emotion in a human relationship is. I only understand simple emotions, such as fear, anger, happiness, and sadness. I cry during sad movies, and sometimes I cry when I see something that really moves me. But complex emotional relationships are beyond my comprehension (Grandin, 2006, 91)

Notwithstanding, some admit, such as Gerland, to not undergoing specific negative emotions:

My lack of envy could also impress other children, and make me seem very clever and sensible. I could watch others turning green with envy in certain situations, without any understanding of that emotion […] I never found out how to covet something someone else had, nor had I any desire to *be* anyone else (Gerland, 1997, 90–91).

In contrast, authors with AS, like James, confessed struggling with the identification of nuanced feelings:

M gives me homework. She asks me to watch a TED Talk on vulnerability by Brené Brown. She gives me a feelings wheel to take away and color in. I’m struck by the sheer number of feelings shown. I just don’t feel any of them. Apart from fear. What does *responsive* feel like? *Valued*? Or *insignificant*? How can anyone feel insignificant when they are at the center of their own life experience? It all seems so complicated and confusing to me (James, 2017, 55–56).

Referring to the cases of individuals with HFA and AS, McGeer supports a version of sentimentalism based not on empathy but on specific emotions (McGeer, 2007). Like us, McGeer criticizes Kennett’s claim that people with ASD have the ability to universalize rules. She argues that they have acknowledged difficulties in both reading other people’s intentions and dealing with dilemmas. However, this behavior is highly variable since ASD covers a wide range of phenotypes. In virtue of their neurodevelopmental disorder, their affective life is peculiar; thus, their choices may be peculiar, depending on specific emotions. The subtheme *feeling basic but not complex emotions* gives credit to McGeer that individuals with ASD have a peculiar affective life. In the examined memoirs, Grandin claims feeling four basic emotions and not understanding ambivalent and mixed feelings, Gerland states she does not comprehend envy and Laura James acknowledges she is not able to feel or cope with more nuanced feelings.^13^ It is also important to note that this, as well, might also be a consequence of the particular social and cultural environment. Currently, programs (Wall et al., 2021) aimed at emotional recognition and enriching the emotional vocabulary of people with ASD are used with positive results. Interestingly, being deprived of certain feelings, individuals with ASD understand the world in a peculiar manner and do not exhibit particular types of behavior in the social environment that a neurotypical shows, as example from Gerland’s statement of the lack of envy illustrates. On the other hand, benefiting by the presence of positive feelings, as shown below, they may be sensitive and helpful in the presence of some external events.

All studied authors reported feeling compassion or concern in the presence of others suffering and these reports were captured by the subtheme *compassion*. They experience distress, a genuine concern for the other person and a desire to help, although in some cases they do not engage in any practical action.^14^

When I was older, I was sorry for Kerstin when she was upset like that. I didn’t understand why she was so shaken, nor what seemed to vibrate inside her, but I could see she was miserable and felt sorry for her […] I liked my sister very much and suffered when she suffered, but it was all incomprehensible and upsetting (Gerland, 1997, 48).

In contrast to McGeer, however, we claim that it is not specific emotions that promote moral actions, rather it is the emotional sharing. All authors referred to feeling compassion on the basis of their experience of sharing suffering. As Gerland commented, she felt sorry for her sister and suffered when she suffered. Our analysis shows that people with autism perform moral actions expressed in behaviors showing concern for others.

All authors report situations in their lives when affective empathy, meaning sharing others’ feelings, such as pain or sadness led them to act in order to reduce others’ suffering or help them. Grandin, for instance, mentions feeling basic emotions such as fear, as a result of empathizing with the cattle, and acting to relieve them^15^ :

I am often asked how can I care about the animals and being involved in slaughtering them […]. However, I am not just an objective, unfeeling observer; I have a sensory empathy for the cattle. When they remain calm I feel calm, and when something goes wrong that causes pain, I also feel their pain. I tune in to what the actual sensations are like to the cattle rather than having the idea of death rile up my emotions. My goal is to reduce suffering and improve the way farm animals are treated (Grandin, 2006, 94).

Another author, Robinson, claims to feel affective empathy only with close ones. The ones that are distant do not move him and do not drive him into any action aimed at helping them:

That’s another kind of empathy. I didn’t have to fix the car. I could have played dumb and she’d never have been any the wiser. I would not have fixed it for anyone besides my mother. But I felt a need to help because a family member was in trouble.

I have what you might call “logical empathy” for people I don’t know. That is, I can understand that it’s a shame that those people died in the plane crash. And I understand they have families, and they are sad. But I don’t have any physical reaction to the news (Robinson, 2008, 32).

As the examples of Grandin and Robinson show, people with HFA and AS engage in helpful practical actions such as working to improve the way farm animals are treated or fixing a car. This observation is supported by previous literature, namely a study by Attwood (2015) pointed out that the (moral) actions of individuals with ASD have a pragmatic character.

We defend a version of pure sentimentalism, not anchored in Kantian precepts also because the theme *affective empathy leads to moral action* shows that sharing feelings with another being is what bolsters moral action. Grandin shared the cattle’s pain, suffering, and sadness and this empathy made her build slaughtering machines better adapted to reduce the anguish of the cattle. In another passage, Robinson mentioned that he only shares feelings with those physically close to him, being his family. When he empathized with his mother’s anxiety, he was able to provide her the help she needed. It is important to emphasize that Robinson helps his mother not because he thinks that is important to help a family member but because he feels the need to help. In contrast, with those being distant as, for example, the injured he saw on TV (whom he has no experience of physical proximity with), Robinson was only capable of understanding the feelings involved. The cognitive empathy he displayed in those circumstances did not move him or motivate moral action. Thus, individuals with HFA and AS affectively resonate with others in empathizing with them rather than by being able to cognitively assess the other’s reasons, intentions, etc. pertaining to their distress. Gerland felt deeply her sister’s despair, although she could not understand it.

Grandin’s and Robinson’s stories point to an experiential dimension of affective empathy. Grandin shares the cattle’s pain and Robinson shares feelings with those physically close to him, which seems to imply that these are felt also at a bodily level. Experiencing the other’s distress primarily at a bodily level can also go along with immediate concern for the other in these cases that is not the result of higher-level cognitive processing or rule-following. Affective empathy can, thereby, move one to action by being based on the own (bodily) experience of the respective emotion by the empathizer, which makes one also directly experience what it is like for the other to be in this situation and, in the case of negative emotions such as distress, be concerned for the other and want to end their distress. In how far the bodily experience of the other’s emotion, as in the cases introduced above, is the sole or primary factor for moral actions can be assessed in further research that focuses on the embodied experience of empathy.

### Temporal Experience

As already mentioned (see section “The Role of Temporal Experience in Emotion”), we adopt a phenomenological notion of temporality that views it as a major structural aspect of human subjectivity that is pre-reflectively present in all experience, actions, and thinking. Time is implicitly felt as the integration of past, present, and future (Fuchs, 2005, 2013; Fuchs and Pallagrosi, 2018) and comprises all three temporal dimensions (Hoy, 2009). It makes possible relating the present moment to times just past and to the moments to come in terms of a general anticipation of the development of events, the consequences of actions, etc.

Temporal experience and the integration of past, present, and future is at the heart of a further theme that emerged in the analysis of the aforementioned autobiographies, namely the relation between the experience of temporality and of empathy and how it affects the moral actions of people with HFA and AS (captured by the theme *difficulty to connect time frames* in our analysis). All of the examined reports included descriptions of difficulties connecting past, present and future in a unified, coherent temporal sequence. This problem took different forms, each of which focused on a different aspect of temporal experience. A reduced ability to anticipate how others act or predict the outcome and consequences of actions one performs focused on the failure to project from the present into a future dimension and was captured by the subtheme *predicting consequences/anticipate others’ behavior*. In addition to this, there were also reports of not being able to envision or imagine the future and experiencing emotions only in response to current events, which would also dissipate once the event was over, and do not extend into the future. These were reflected in the analysis by the subthemes *imagining the future* and *feeling emotions at a particular time frame*. Based on the reports of these alterations of temporal experience, it appears that people with HFA and AS are emotionally engaged with the present, having difficulties imagining what a future would be like. Half of them point out they are not emotionally moved by past events but only by present ones. The empathic arousal mode of people with HFA and AS shapes their moral actions by introducing constraints to the expression of moral actions.

Like most of the authors in this sample, Willey did not anticipate others’ behavior. When she saw a frightening stranger in a friendly environment, she did not predict the danger that might come^16^ :

Thankfully, miraculously, a male student I had never known to be early before, came into the class and quickly and confidently walked to my side so that he was wedged between the man and myself. For some reason, the student’s closeness to me did not offend me, but it did bother the man. In the blink of an eye, he disappeared out the door. When the man was gone from the room, I remember the student ask me if I was okay, if I needed anything, if the man had hurt me. I remember remaining very calm, almost wondering why he was so concerned, then I remembered the man’s smell, his violating my personal space. Then, I knew I should have been afraid. I knew I had made a terrible error in judgment. I knew I had just been very, very lucky (Willey, 1999, 70–71).

Another author, Robinson, did not predict the consequences of his words when he tactlessly voiced the truth:

A few years later, Uncle Bob decided to get married again. At the wedding, I said the first thing that came to mind, in typical Aspergian fashion: “Uncle Bob, how many times do you have to get married before you stay married?” I don’t remember what he said, but I remember the result: I wasn’t invited to his next wedding (Robinson, 2008, 253–254).

Robinson did not think twice when he commented on his uncle’s marriage. He failed to see that honesty can sometimes transform itself into insolence. We suggest that individuals with ASD fail to perform well when it comes to moral stances that require diplomacy, negotiation, and subtler adequacy of moral principles to practical life because they fail to anticipate the consequences of their actions or words due to a difficulty of temporal projection, as other authors have corroborated (Sturrock et al., 2022). Another typical case are white lies – something individuals with ASD are hardly able to perform (Williams et al., 2018). They value truth but are not prudent to observe the best circumstance to voice it.

The perception of temporality is not restricted to reading correctly others’ behavior and predicting their subsequent actions. People with ASD have trouble not only in imagining others’ future but also their own. Most of the authors report that they have difficulties envisioning the future and conceiving it as different from the present. This may result in repetitive actions and inconsiderate behavior, as Prince-Hughes puts it^17^ :

My concept of “the future” was, for all intents and purposes, completely underdeveloped. If something was one way at the moment, I couldn’t imagine it any other way. If a woman desperately wanted my company at a given point in time, I could never conceive of it being any other way. This same inability to see the future as developing into something different from the present was a further stumbling block in my education and other areas of growth. This lack of future concept may be responsible for some of the poor impulse control that many autistic people struggle that can make relationships so difficult (Prince-Hughes, 2004, 84).

The difficulty to imagine the future might prevent individuals with ASD from recognizing when it is warranted or appropriate to act as they anticipate the future as merely a uniform continuation of the present, which does not change in any way. Thereby, they might not anticipate developments or changes of the present state that prompt a reaction or action in the future on their part. Prince-Hughes assumed that if at some time a woman wanted her company, she would want it forever. She expected a steady continuation of the current state and was not capable of conceiving an alternative scenario.

More than half of the authors, including all of those who were labeled HFA and two of whom were diagnosed with AS, reported feeling exclusively event-related emotions. Other social partners, at the time ill-prepared to understand autism, were not able to support persons with ASD to also emote toward past or future events. Probably, they would have failed to do so, too, if they had known their conditions better. Furthermore, since individuals with autism lack flexibility in adapting their emotional responses to different circumstance but rather continue to express them in the same way, they cannot be called resilient. They are neither haunted by events of the past nor anticipate future events and emote about those.^18^ As Schneider voices:

I had mentioned earlier that I do experience the survival-type emotions: fear and anger. So do the NT [neurotypicals], but, for them, like their other emotions, they are persistent; mine are pure situational. Fear does not become phobia, nor does anger turn into hatred. When the incident that provoked those emotions passes, so does the feeling. I carry neither torches nor grudges (Schneider, 1999, 102).

Or Grandin who comments:

When I get angry, it is like an afternoon thunderstorm; the anger is intense, but once I get over it, the emotion quickly dissipates. I become very angry when I see people abusing cattle, but if they change their behavior and stop abusing the animals, the emotion quickly passes (Grandin, 2006, 90).

In some cases, people with ASD are even unable to link their present emotional circumstances with past events prompting them to impulsive behavior, as the examples provided by Schneider (1999) and Grandin (2006) show.^19^ From this we conclude that the emotions mobilizing moral actions are situated in the present, they are not linked to future emotional outcomes. As a result, the moral actions exhibited due to these constraints is atypical. According to Hoffman (1984), complex moral actions occurs when we recall past events and link them to present emotions to anticipate future events. The empathic arousal mode experienced by people with HFA and AS corresponds to the stage that Hoffman called *conditioning and motor mimicry* (Hoffman, 1978), which is an immediate and non-cognitive experience of an emotion. This is also mirrored in how they empathize with others, namely by directly experiencing distress when observing others in distress. As Gerland (1997, 48), for example, reported “(I) suffered when she suffered.” The same is valid for Grandin (2006) who explicitly emphasizes that even intense emotions are only present-related and do not extend into the future. This dominance of the present is also mirrored in the primacy of affective empathy. Experiencing the other’s distress or suffering is confined to the present moment of feeling it rather than empathizing in terms of a cognitive process. Consequently, people with HFA and AS did not suffer from blame over inaction or anticipatory guilt, signs of moral agency associated with Hoffman’s other stages of empathic arousal mode. Like Schneider puts it “I carry neither torches nor grudges” (Schneider, 1999, 102). This phenomenon may also be a consequence of inadequate support that these people received during their development with respect to their disorder. The difficulty to anticipate future situations and how to deal with them might put them more at risk of getting involved in dangerous situations, as in Willey’s case, or of finding themselves in disagreeable circumstances, as the example of Robinson shows.

It might be argued that the difficulties people with ASD encounter in managing and following temporal sequences described in the last theme would naturally lead to impulsive behavior. Thus, it would not be surprising if moral actions were motivated by momentary impulses. To this objection, it can be answered that impulsive behavior derived solely from temporal impairments does not lead to moral action. For instance, gamblers, who are driven by impulsive choices and react very quickly, may harm themselves and others in virtue of their actions (Grecucci et al., 2014). It is the disturbance in temporal processes, connected with specific empathic processes, in people with ASD that engage them in peculiar moral actions. For instance, by following Robinson’s example in theme (2), we could observe that Robinson’s (2008) impulsive behavior could have led him ‘to play dumb’ when his mother needed his help. However, because he was engaged in emotional sharing with his mother, he tried to help her.

## Moral Agency, Rules, and Temporality

This paper discusses two approaches to moral agency in persons with mild forms of autism, the rationalist and the sentimentalist perspectives. The rationalist view argues that moral actions are based on reasons (McGeer, 2007). In particular, as Kennett (2002) has proposed, persons with ASD stick to universal rules that are supposed to govern the moral actions of everyone. As we have seen in the subthemes of the main theme *Following Rules* (*difficulty to generalize; norms only apply to themselves; use norms of as technique to thrive*), at least some people with mild forms of autism are unable to extend the rules governing moral actions beyond their own personal sphere to a community, in other words, they are unable to universalize these rules. Moreover, altered experience of temporality, reflected in the subthemes *predicting consequences/anticipate others’ behavior* and *imagining the future*, contributes to their lack of adherence to universal rules. By having difficulty anticipating future moments and accordingly also the consequences of events or actions, also rules seem bound only to the present situation and those involved in it. Universalizing them is, thus, not possible as one fails to project them to other situations at other times.

On the other hand, the sentimentalist view states that our moral actions are based on empathic feelings (McGeer, 2007). A proponent of this vision, Bollard (2013), argues that although moral agency is rooted in emotions, the role of emotions is restricted to choosing a particular set of norms to behave. It is true that Bollard does not consider the norms that persons with ASD follow to be universal. However, we contest that in a sphere so complex as in the domain of moral issues, people with ASD are driven even by particular norms. Bollard’s view requires that emotions follow a temporal sequence encompassing past actions and future projections that characterize also particular norms. As we saw in the subtheme *Feeling emotions at a particular temporal frame*, emotions in people with ASD operate in the present. We do not deny that people with ASD build particular norms to act, as the example of Williams painting graffiti at the wall illustrates, but they are certainly not based on emotions and not reserved to the moral domain. As situated in the present, emotions lead to impulsive actions and not to strategic thinking, as depicted by subtheme *Affective empathy leads to moral actions*. The subthemes of the theme *Feeling a specific set of emotions*, illustrate the peculiar emotional life of people with ASD, which coupled with an impaired temporality, leads them to perform moral actions in a peculiar impulsive way. Experiencing predominantly the present moment and having a different sense of temporal projection, one is more likely to feel prompted or motivated to act at the very moment and not so much in view of anticipating the future consequences of presently unfolding events.

The difficulty of generalizing rules, the specific range of affective experience, and the disturbance of temporal experience, moreover, are in line with understanding ASD in relational or externalist terms as a diminished fluency in engaging with so-called social institutions (Roberts et al., 2019). ASD, on this view, is constituted by both disruptions of embodied practices essential for social interactions and by the failure of responding appropriately and adequately to individuals with ASD that originate from common interactional patterns and expectations inherent in social practices and institutions. Autism, in relational or externalist terms, is (at least partly) determined by contextual and situational factors that, moreover can be best addressed by studying autobiographical narratives from a phenomenological perspective that focuses on the individual lived experience embedded in the realm of social interactions.

## Limitations

It is important to note that our study faces some limitations. Firstly, as the sample we studied includes a larger number of autobiographies authored by women, and ASD is characterized by a higher prevalence in male populations (Werling and Geschwind, 2013), our sample might not be very representative. The authors of all examined autobiographies are Caucasian, which might have impacted culturally and socially transmitted aspects of empathy and emotions.^20^ Another restriction concerns the definition of autism spectrum disorder, which has been subjected to discussion and revision over the years. In this study, we adopted the definitions of HFA and AS, now outdated, because they were of common usage at the time the majority of the autobiographies were written. Autism spectrum disorder is a complex and heterogeneous disorder with different phenotypes and the fact that we based our study on autobiography selection preclude us from a fine-grained analysis of the type of autism that authors exhibit. Further scientific studies should carefully address the participants’ diagnoses of autism by recurring to interviews and questionnaires. Therefore, we consider that no firm clinical conclusions can be drawn from the study and that the generalization of the results is not possible for practical purposes. However, we propose that this study can provide a theoretical framework for drawing further scientific studies of how people with ASD make moral decisions.

In fact, the limitations pointed out do not diminish the value of this study, at least from a philosophical point of view. Previous studies such as the investigation of Kennett (2002) and Bollard (2013) also support their argumentation with autobiographical reports by individuals with ASD. However, these researchers recurred only to some examples taken from the autobiographies to sustain their views, without using methodological procedures, like qualitative analysis, to systematically study autobiographical material. As far as we know, this is the first study in the philosophical realm to support the argumentation with an analysis of autobiographies by individuals with ASD. Following a method to study the autobiographies and supporting the philosophical argumentation with the results from such a method constitutes a major advance in relation to previous literature.

Finally, it is important to note, though, that our findings only feed into the sentimentalist-rationalist debate concerning individuals with ASD, commonly considered an atypical population, thus limiting interpretability and extrapolation of the conclusions to a general public. Nevertheless, even considering these restrictions, we may admit that the particular debate discussed in this study leads us to pose questions about the neurotypical bases of moral agency. The motive for such consideration is that the models of disorder/illness, in general, allow us to see how human beings behave when a deficit or an excess of a characteristic is present. Thus, we can learn more about that particular feature or, by contrast, about healthy human behavior.

## Conclusion

The goal of this paper was to investigate the mechanisms of moral agency in persons with ASD, by finding arguments for the origin of their moral agency, supporting either the sentimentalist or the rationalist view of the debate. An interpretative phenomenological analysis of autobiographies authored by individuals with ASD revealed a relationship between sharing affective experience and moral actions, identified as being concerned for others’ suffering and engaging in helping behavior. Contrary to a rationalist position, people with ASD do not morally act by following universal rules [contrary to Kennett (2002)]. Contrary to the claims of traditional sentimentalist approaches, though, individuals with ASD do not derive norms on the basis of affective experience and act morally on the basis of these [contrary to Bollard (2013)]. People with ASD are moved to moral actions by sharing the affective experience of others. In addition to this, we also uncovered a relationship between the experience of temporality, empathy, and moral agency Individuals with ASD tend to engage in empathizing with presently experienced emotions of others, which can be related to a general pattern of focusing on the present and not engaging in anticipation of the future and rumination over the past. As a previous study suggested, individuals with ASD live in an interrupted present (Vogel et al., 2019). Compared to neurotypical individuals, who might resort to cognitive empathy as well, the authors of the memoirs showed a preference for affective empathy, which is in line with previous literature (Smith, 2009). It was also reported in the investigated autobiographies not being prone to experiencing at least some negative emotions such as envy and greed. Consequently, individuals with ASD do not engage in envy- or greed-driven behaviors, as also previous studies have shown (Shamay-Tsoory, 2008).

In line with previous case studies of people with ASD, we have shown that affective empathy can lead to a moral response, while previous case studies of people with psychopathy, in contrast, revealed that reason alone (with strong emotional deficits) is incapable to induce moral actions (Bollard, 2013). Thus, at least we can sustain that affective empathy has a role in moral agency.

Future work could investigate whether the interference of a rational process delays or even inhibits the moral response in a neurotypical individual, who accesses both cognitive and affective empathy, follows action sequences and does not have temporal impairments. Additionally, it should be further elucidated whether affective empathy, in healthy persons, by itself constitutes the complete origin of moral agency.

The fact that people with ASD in this study did not receive an appropriate response to their disorder in the course of their lives may let us suppose the impact that a society unprepared to deal with ASD might have in shaping the social answers of these people with ASD. They usually react to the external world recurring only to their internal resources and the way they experience the world is constrained by the lack of appropriate responses to their inner problems. It would be interesting in the middle of XXI century to conduct a similar study as this one and to verify if the social replies, shaped by years of treatment and a comprehensive social environment would be the same.

## Data Availability Statement

The original contributions presented in the study are included in the article, further inquiries can be directed to the corresponding authors.

## Author Contributions

SC contributed to the conceptualization, the methodology, the writing, and investigation related to this study. SB and ED contributed to the methodology of the study and writing of the manuscript. AP-Y contributed to writing and supervision. AS contributed to the conceptualization and supervised the study, and writing of the manuscript. All authors contributed to the article and approved the submitted version.

## Conflict of Interest

The authors declare that the research was conducted in the absence of any commercial or financial relationships that could be construed as a potential conflict of interest.

## Publisher’s Note

All claims expressed in this article are solely those of the authors and do not necessarily represent those of their affiliated organizations, or those of the publisher, the editors and the reviewers. Any product that may be evaluated in this article, or claim that may be made by its manufacturer, is not guaranteed or endorsed by the publisher.

## Footnotes

1 We base our notion of moral agency on Husserl’s definition, according to which the desire of realizing something deemed “good” directs a subject to put it in practice. Furthermore, it may be the object of a deliberation or not (Drummond and Timmons, 2021). We use the terms “moral agency” and “moral actions” interchangeably.

2 The term “spectrum” has been used since the symptoms’ presentation and severity, as well as the skills and level of functioning of people with this diagnosis is diverse. ASD has an early-onset and its symptoms can change with development and may be masked by compensatory mechanisms, although they still cause significant impairment in the adults’ presentation (American Psychiatric Association, 2013). Starting from the DSM V, AS has been included in the ASD, so that the distinction between HFA and AS is no longer available. Since the majority of the patients considered in this research were diagnosed when that distinction was used, we decided to keep it. The main difference between the two terms is that people classified with HFA have a delay in language development, while people with AS do not.

3 Some authors, like Hodges and Myers (2007), include the emotions of compassion and distress in their definition of empathy, while others (De Vignemont and Singer, 2006; Paulus et al., 2013) state that although affective empathy is distinct from these feelings, it prompts the empathizer to feel them and to act accordingly. In this paper, we go for the second option.

4 Psychopathy is a condition characterized by anti-social behavior, insensibility to the distress of others and lack of remorse and guilt. Psychopathy itself does not fit in a mental disorder category but it is commonly captured by the diagnosis of Antisocial Personality Disorder (American Psychiatric Association, 2013, p. 659).

5 “I have to develop a separate translation code for every person I meet […] does it indicate an uncooperative attitude if someone doesn’t understand information conveyed in a foreign language? Even if I can tell what the cues mean, I may not know what to do about them. The first time I ever realized someone needed to be touched was during an encounter with a grief-stricken, hysterically sobbing person who was in no condition to respond to my questions about what I should do to help. I could certainly tell he was upset. I could even figure out that there was something I could do that would be better than nothing. But I didn’t know what that something was” (Sinclair, 1992).

6 It is important to note that the alterations in temporal experience in different psychopathological conditions have been widely researched especially in phenomenological accounts such as Ratcliffe (2012); Fuchs (2013), and Stanghellini et al. (2016) among others. Pokropski (2015) directly addresses the role of temporality in social cognition. However, the qualitative aspects of temporal experience so much pursued in phenomenological research have been overlooked in empirical research, which gave priority to duration estimation studies (Moskalewicz and Schwartz, 2020). Therefore, the issue of lived time in people with ASD is under-studied and, as a result of the prevalent studies, some authors advise to be cautious when claiming direct relationships between people with ASD’s time perception and their behavioral and emotional experience (Falter and Noreika, 2011; Vogel et al., 2018). In the absence of methodological studies, some hypotheses launched by phenomenology remain speculative.

7 It is important to acknowledge that this subtheme was first mentioned in the paper “Who really needs a ‘theory’ of Mind” by Williams (2009). Williams, however, uses the subtheme in a context different from ours. In fact, Williams aims to argue against the Theory of Mind.

8 Gerland (1997, 117–118), Willey (1999, 107), Prince-Hughes (2004, 84), Grandin (2006, 158), and Robinson (2008, 89) describe similar phenomena.

9 Similar descriptions are also mentioned by Schneider (1999, 95), Willey (1999, 44), Williams (1999, 76), Lawson (2000, 17), Prince-Hughes (2004, 156), Grandin (2006, 108–109), and James (2017, 139).

10 Below, in the sub-theme *norms only apply to themselves*, a quote can be found where Gerland (1997) is called pampered by her peculiar social conduct. The norms that people with ASD like Robinson and Gerland chose to follow in order to thrive reflect also the failure of the social environment to recognize their difficulties and to adjust to their peculiar situations. Unable to function well in a world with universal rules, such as “No one should paint graffiti on the walls of any building,” as in Williams’ case, and not being understood by a world that does not adapt to them, they are left to their own devices. Thus, they create their own values and norms as they do not find logic in the outside world, such as in Kammer’s case. We may risk to say that if a more caring environment was provided, they would perhaps create and follow more norms that do not concern themselves only but also apply to others.

11 Further passages describing this subtheme can be found in Willey (1999, 45), Williams (1999, 29), Lawson (2000, 16), Prince-Hughes (2004, 53–58), Grandin (2006, 108), Robinson (2008, 239–240), and James (2017, 46).

12 Further descriptions of experiencing specific emotions were found in Schneider (1999, 25), Willey (1999, 82–83), Williams (1999, 36), Lawson (2000, 65), and Robinson (2008, 255).

13 A discussion whether the affective life mentioned might reflect difficulties in classifying emotions rather than an absence of these emotions goes beyond the scope of this paper.

14 Further descriptions of feeling compassion for others can be found in Schneider (1999, 67), Willey (1999, 62), Williams (1999, 40), Lawson (2000, 82), Prince-Hughes (2004, 65), Grandin (2006, 176), Kammer (2007, 29), Robinson (2008, 31), and James (2017, 139).

15 Descriptions of experiences of affective empathy, which leads to moral actions can be found also in Gerland (1997, 213), Schneider (1999, 80), Willey (1999, 125), Williams (1999, 37), Lawson (2000, 88), Prince-Hughes (2004, 170), Kammer (2007, 13), and James (2017, 135).

16 Descriptions of a failure to predict consequences and anticipate others’ actions can be found also in Gerland (1997, 85), Schneider (1999, 67), Williams (1999, 50), Lawson (2000, 73), Grandin (2006, 113), and James (2017, 163).

17 Further reports of an inability to imagine the future or a future different from the present can be found in Gerland (1997, 156), Willey (1999, 52), Williams (1999, 64), Grandin (2006, 94), Robinson (2008, 192), and James (2017, 111).

18 Gerland (1997, 85), Willey (1999, 89–90), Williams (1999, 92), and James (2017, 57) also describe this phenomenon.

19 It is important to note here that the phenomenon of projecting or linking presently experienced emotions to future situations or their outcomes in the future differs significantly from emotional resilience. While emotional resilience refers to the ability to adapt to stressful situations, the phenomenon we are considering is not concerned with the impacts of negative situations and emotions, but solely with a projection or extrapolation of presently felt emotions to future situations. Both Schneider and Grandin show us their usual emotional processing. They do not engage in strategies to have better outcomes in interpersonal relations, such as the strategy of camouflage (pretending certain types of behavior to fit in society) lets us suppose (Szatmari, 2018).

20 Cassels et al. (2010), for instance, have discovered cultural differences in affective empathy between Western and East Asian cultures by examining empathy in bicultural individuals. According to them (Cassels et al., 2010) both family and community impact affective empathy.
